# Implicit Theories of Intelligence and Achievement Goals: A Look at Students’ Intrinsic Motivation and Achievement in Mathematics

**DOI:** 10.3389/fpsyg.2021.593715

**Published:** 2021-02-22

**Authors:** Woon Chia Liu

**Affiliations:** National Institute of Education, Nanyang Technological University, Singapore, Singapore

**Keywords:** implicit theories, mindsets, achievement goals, interest, intrinsic motivation, mathematics achievement, Singapore, lower-progress students

## Abstract

The present research seeks to utilize Implicit Theories of Intelligence (mindsets) and Achievement Goal Theory to understand students’ intrinsic motivation and academic performance in mathematics in Singapore. 1,201 lower-progress stream students (596 males, 580 females, 25 missing data), ages ranged from 13 to 17 years (*M* = 14.68 years old, *SD* = 0.57), from 17 secondary schools in Singapore took part in the study. Using structural equation modeling, results confirmed hypotheses that incremental mindset predicted mastery-approach goals and, in turn, predicted intrinsic motivation and mathematics performance. Entity mindset predicted performance-approach and performance-avoidance goals. Performance-approach goal was positively linked to intrinsic motivation and mathematics performance; performance-avoidance goal, however, negatively predicted intrinsic motivation and mathematics performance. The model accounted for 35.9% of variance in intrinsic motivation and 13.8% in mathematics performance. These findings suggest that intrinsic motivation toward mathematics and achievement scores might be enhanced through interventions that focus on incremental mindset and mastery-approach goal. In addition, performance-approach goal may enhance intrinsic motivation and achievement as well, but to a lesser extent. Finally, the study adds to the literature done in the Asian context and lends support to the contention that culture may affect students’ mindsets and adoption of achievement goals, and their associated impact on motivation and achievement outcomes.

## Introduction

Human behavior is complex, and no single psychological theory can explain all aspects of human motivation and achievement (Roberts, 1992). Nonetheless, several theories, for example, Implicit Theories of Intelligence (Dweck, 2000) and Achievement Goal Theory (Elliot, 1999), have revealed important determinants of motivation and achievement in education. The present study seeks to utilize these two theories to understand the learning engagement and academic performance in mathematics of lower-progress students in Singapore.

Singapore has an educational system where students are streamed nationally into different ability streams based on their academic performance in the Primary School Leaving Examination (PSLE) at the end of Year 6 (approximately 12 years old). The three streams are the Express stream, the Normal (Academic) stream, and the Normal (Technical) stream. The Express stream generally consists of students in the top 65% of the secondary school cohort, while the Normal (Academic) and Normal (Technical) streams consist of the remaining 35% who qualify for secondary school. As such, the Express stream is considered the higher-progress stream, while the Normal (Academic) and Normal (Technical) streams, collectively known as the Normal stream, are considered the lower-progress stream. By identifying the determinants of lower-progress students’ motivation and achievement, this study hopes to offer suggestions for intervention that can help engage this group of students and promote learning engagement and academic performance in the classrooms.

Considering that socialization plays a role in shaping an individual’s belief system, it is conceivable that students in Singapore and other Asian countries may view ability, learning, and achievement differently compared to their Western counterparts. There is evidence to suggest that more collectivist societies might encourage students to value the learning process over academic achievement and focus less on individual results (Costa and Faria, 2018). In contrast, a more academically competitive society in Europe might influence the students’ perspectives of intelligence and lead them to prioritize individual outcomes and to value positive assessment over knowledge (Elliott and Dweck, 1988; Robins and Pals, 2002). With a lack of studies on mindsets and achievement goals in the Asian context, this study will also add to the literature and provide insights into Asian students’ mindsets and adoption of achievement goals, and their associated impact on motivation and achievement outcomes.

### Intrinsic Motivation

Intrinsic motivation is defined as activities done “for their own sake” or for their inherent interest and enjoyment (Deci and Ryan, 2000). It is deemed to be responsible for most of human learning across the life span, in contrast to externally mandated learning and instruction (Ryan and Deci, 2017). It is seen as an important consideration when examining participation in tasks that require perseverance and sustained levels of effort (e.g., Stanko-Kaczmarek, 2012). It has been found to play a significant role in student engagement (Froiland and Worrell, 2016) and school achievement (e.g., Taylor et al., 2014) and is frequently studied as an outcome of Achievement Goal Theory and Implicit theories (Cury et al., 2006). In Singapore, intrinsic motivation (or interest) is recognized as an important factor in enhancing lifelong learning in schools (Wang, 2017) and is included as a key outcome of the current study.

### Implicit Theories of Intelligence

Implicit theories—or mindsets—about human abilities are important for academic learning. They form a belief system that triggers particular motivations, leads to different learning pathways, and shapes how individuals interpret and understand their learning experiences. Dweck and her colleagues (Dweck et al., 1995; Dweck, 2000) proposed the Implicit Theories of Intelligence to explain how individuals’ implicit theories (mindsets) set up both a motivational and cognitive framework that colors the individuals’ views of and responses to learning engagement and achievement.

According to Dweck and her colleagues (Dweck et al., 1995; Dweck, 2000), human mindsets can be categorized in two forms—incremental (growth) and entity (fixed) mindsets. Individuals with incremental mindsets—the incremental theorists—believe that intelligence is malleable and can be increased through effort. Incremental theorists are concerned with achieving mastery through learning. They tend to use performance outcomes as feedback to reflect on their task commitment and learning strategy. By contrast, individuals with entity mindsets—the entity theorists—believe that intelligence is fixed and cannot be changed. Entity theorists tend to judge their fixed level of intelligence based on performance feedback. They would conclude that they are smart if they perform well on academic tasks, and not smart if they perform poorly on these tasks. When entity theorists receive negative performance feedback, they tend to make sweeping generalizations about their lack of ability, give up prematurely, and show debilitation over time.

Research has provided evidence that mindsets predict achievement (e.g., Romero et al., 2014; Müllensiefen et al., 2015; Costa and Faria, 2018). Generally, research examining the different response patterns of students’ mindsets had found that incremental mindsets, relative to entity mindsets, tended to be associated with better academic achievement (e.g., Blackwell et al., 2007; Burnette et al., 2013; Romero et al., 2014; Bostwick et al., 2017). Nonetheless, Costa and Faria (2018) found that culture was a moderator of the relationships. Using a meta-analytic approach, they established that incremental mindsets were associated with higher levels of students’ achievement in Asia, Oceania (Australia), and at the limit of significance in North America but were not significant for Europe. In contrast, entity mindsets were not significantly associated with achievement in Asia but were negatively associated with student achievement in North America and positively associated with student achievement in Europe.

In addition, Dweck and her colleagues (Dweck and Leggett, 1988; Dweck, 2000) proposed that mindsets are the antecedents of achievement goals. This is because a mindset forms a belief system that triggers a particular achievement goal. In the next section, we will discuss the concept of achievement goals.

### Achievement Goals

The achievement-goal approach has generated a huge amount of conceptual and empirical work over the last 40 years with different perspectives and positions on how to operationalize the construct (e.g., Korn and Elliot, 2016). Nevertheless, there is a general consensus that achievement goals are related to the reasons for behaviors in achievement situations (e.g., Dweck, 1989; Nicholls, 1989), and the standards of reference for evaluating one’s competence and success (Elliot, 1997).

In the initial dichotomous model of achievement goals proposed in the 1980s, Nicholls (1984), among many others, conceptualized achievement goals according to the *focus of competence*, and two ways of defining success—attainment of mastery (self-referenced success) and outperforming others (other-referenced success). Individuals who pursue the mastery goals are concerned with the development of ability. They are likely to seek achievement by developing competence and acquiring knowledge through effortful learning (Murphy and Alexander, 2000). These individuals define success in terms of the extent of mastery of the learning task (Pintrich, 2000). They are more likely to appreciate the intrinsic value of learning, see effort as the main factor defining their success, and evaluate their level of competence and learning based on self-established standards of achievement. In contrast, individuals who pursue performance goals seek “to gain favorable judgments and avoid negative judgments of one’s competence, particularly if success is achieved through a minimum exertion of effort” (Murphy and Alexander, 2000, p. 28). These individuals define success in terms of their ability or performance relative to others (Pintrich, 2000). They judge their competence and sense of self-worth through whether they can outperform others or achieve their targets with less effort on norm-referenced standards set by external authorities. In general, mastery goals are associated with more adaptive outcomes, while performance goals are linked with less adaptive outcomes (see Elliot, 2005).

In the 1990s, achievement goal theorists began to include an additional component of competence, that is, the *valence of competence*, in their conceptual work (Elliot and Harackiewicz, 1996). This development kept mastery goals intact but divided performance-based goals into performance-approach and performance-avoidance, resulting in a three-goal trichotomy. A few years later, Elliot (1999) expanded the concept by proposing that both mastery and performance were fully crossed with approach and avoidance. In other words, individuals pursuing mastery goals may be motivated to approach mastery or to avoid lack of mastery. Likewise, individuals pursuing performance goals may be motivated to approach good performance or to avoid poor performance. This conceptualization yielded a 2 × 2 model featuring four types of achievement goals: mastery-approach, mastery-avoidance, performance-approach, and performance-avoidance goals (Elliot and McGregor, 2001). Subsequent research led to the differentiation of mastery goals into task-based and self-based standards (Elliot et al., 2011). With three different standards to evaluate competence, that is, task-based, self-based, and other-based, fully crossed with approach and avoidance, a 3 × 2 achievement goal model was obtained.

In this study, the 2 × 2 model featuring mastery-approach, mastery-avoidance, performance-approach, and performance-avoidance was adopted to draw comparisons to previous work exploring achievement goals of students in the Asian context. Using the 3 × 2 model will preclude any comparison to earlier studies.

### Implicit Theories, Achievement Goals, Intrinsic Motivation, and Achievement

The four achievement goals of the 2 × 2 model are conceptually orthogonal and independent and are associated with different achievement and affective outcomes. Mastery-approach goals are largely linked to adaptive outcomes, such as intrinsic motivation and enjoyment (e.g., Fox et al., 1994; Biddle et al., 2003) and positive affect (e.g., Ntoumanis and Biddle, 1999). In comparison, the consequence of adopting performance-approach goals is more debatable (e.g., Midgley et al., 2001; Harackiewicz et al., 2002a). They are associated primarily with a positive but truncated set of positive outcomes (Elliot, 2005) and may be adaptive in the sense of promoting graded academic performance (e.g., Elliot and Church, 1997; Elliot and McGregor, 1999; Church et al., 2001; Harackiewicz et al., 2002b; Liu et al., 2009; Jang and Liu, 2012).

Although there was initial skepticism regarding mastery-avoidance goal, empirical evidence has supported the existence of this goal and suggested that mastery-avoidance goal is prevalent in achievement settings (e.g., Van Yperen, 2006; Liu et al., 2009; Jang and Liu, 2012). Specifically, mastery-avoidance and performance-avoidance goals are generally associated with less adaptive outcomes, such as low performance, low intrinsic motivation, disorganization, worry, and emotionality (e.g., Elliot and Church, 1997; Middleton and Midgley, 1997; Elliot and McGregor, 1999, 2001; Church et al., 2001; Wolters, 2004; Van Yperen et al., 2009).

In a meta-analysis of 98 papers with a sample size of 33,983 participants on achievement goals and achievements across work, sports, and education, Van Yperen et al. (2014) affirmed that both approach goals (mastery and performance) are related to positive performance attainment, whereas both avoidance goals (mastery and performance) are negatively associated with performance attainment. However, they found that nationality moderated the relationships between mastery-based goals and achievements. Most notably, mastery-approach goal seems to be more beneficial among Asian and “other” samples in comparison to US/Canadian and European samples, whereas mastery-avoidance goal seems to be more negatively related to achievement for Asian and US/Canadian samples in comparison to European and “other” samples. The finding underlined the importance of acknowledging the role of culture in motivational research (Pintrich, 2003). There are, nevertheless, limited studies that had interpreted their findings in light of the specific world region in which they had been derived (Bardach et al., 2019), and even fewer studies had been done in the Asian context or with Asian participants. For instance, Van Yperen and colleagues noted that the majority of the participants in the 98 studies were of US or Canadian nationality (59.0%), followed by European (23.0%), with only 10.8% Asian, and 7.2% other nationalities. Clearly, more research is needed in the Asian context to clarify or confirm the findings. It is too simplistic to assume that the findings for US/Canadian or European samples can be generalized to Asian participants.

In the Singapore context, using an intra-individual cluster-analytic approach to examine goal profiles, researchers established that students who were high in all four goals, that is, high in mastery-approach, mastery-avoidance, performance-approach, and performance-avoidance goals, and those who were only high in mastery-approach goals tended to be associated with positive psychological characteristics and outcomes (e.g., Liu and Wang, 2005; Wang et al., 2007; Liu et al., 2009). Additionally, Jang and Liu (2012) found that students who were high in all four goals had high mathematics performance but also high anxiety and moderate boredom. In contrast, students who were only high in mastery-approach and low in mastery-avoidance profile reported high mathematics performance, high enjoyment, low anxiety, and low boredom. It is noteworthy that higher-progress students were overrepresented in the more adaptive clusters, whereas lower-progress students were overrepresented in the less adaptive clusters.

As mentioned earlier, the adoption of achievement goals may be related to the mindsets that individuals hold. Dweck and her colleagues (Dweck and Leggett, 1988; Dweck, 2000) proposed that mindsets form a belief system which may orient the individuals toward particular motivational goals which may in turn lead to different learning pathways. More specifically, mastery goal is associated with having an incremental view of intelligence, and performance goal is linked with an entity view of intelligence (Dweck and Leggett, 1988; Burnette et al., 2013). In addition to finding that incremental theorists have the tendency to adopt mastery goals and demonstrate mastery-oriented responses to academic setbacks, Dweck (2000) further found that for entity theorists with higher confidence in their intelligence, they were likely to adopt performance-approach goals, while those with lower confidence were likely to adopt performance-avoidance goals. Burnette et al. (2013) also revealed that the positive association between mindsets and mastery goals is stronger for mastery-approach goals than for mastery-avoidance goals. In comparison, the negative association between mindsets and performance goals is stronger for the performance-avoidance goal than for the performance-approach goal.

In the Singapore context, Liu and Wang (2005) found that students who were high in all four goals had a significantly higher entity mindset than students who were only high in mastery-approach goals, although both clusters tended to be associated with positive psychological characteristics and outcomes. In the domain of sports, studies (e.g., Wang and Biddle, 2001, 2007; Biddle et al., 2003) have shown that both the incremental mindset and mastery goals are linked to intrinsic motivation and adaptive motivational outcomes. In contrast, entity mindset and performance goals are associated with low intrinsic motivation, low perceived competence, and maladaptive learning outcomes. In line with Dweck’s (2000) finding, Wang et al. (2009) established that perceived competence moderated the relationships between mindsets and avoidance goals in the domain of sports. Specifically, entity beliefs predicted performance-avoidance goals when perceived competence was moderately low but not when high. Likewise, incremental beliefs predicted mastery-avoidance goals when perceived competence was moderately low but not high.

Taken together, the findings from the abovementioned studies suggest that there is an association between mindsets and achievement goals. The two mindsets relate to the 2 × 2 achievement goals differently, and the two mindsets and the 2 × 2 achievement goals relate to learning outcomes differently, perhaps with different associations in different cultural contexts.

### Rationale of Study and Hypotheses

The present study utilizes Implicit Theories of Intelligence (Dweck, 2000) and 2 × 2 Achievement Goal Theory (Elliot and McGregor, 2001) to understand the intrinsic motivation (interest) and academic performance (score) in mathematics of lower-progress students in Singapore.

This study focuses on lower-progress students because empirical studies have suggested that in general, lower-progress students relative to higher-progress have motivational related issues such as lower intrinsic motivation and self-determination (e.g., Chow and Yong, 2013; O’Shea et al., 2017), and lower self-esteem, more negative self-concepts, and poorer social adaptation (Safree et al., 2009). Studies in Singapore have indeed found that lower-progress students had significantly lower mathematics achievements and mathematics self-concept than higher-progress students (Liem et al., 2015). In addition, lower-progress students were overrepresented in more maladaptive clusters that had lower intrinsic motivation and mathematics performance and higher anxiety and boredom compared to higher-progress students (Jang and Liu, 2012).

It will be recalled that perceived competence can moderate the relationships between mindsets and goals (e.g., Dweck, 2000; Wang et al., 2009). Since stream membership is an explicit label of ability and a reflection of the students’ academic competence, it is tenable that the relationships between mindsets, achievement goals, intrinsic motivation, and academic performance may not be the same for higher‐ and lower-progress students. As such, it is important that a theoretically driven research to examine lower-progress students’ motivation be conducted to guide interventions.

Considering the scarcity of research in the Asian context as compared to research in the US, Canada, and Europe, this study will also be able to shed light on the relationships between the aforementioned constructs in a different cultural context and hence expand our knowledge base on the interaction between mindsets and achievement goals on learning outcomes.

Additionally, this study is premised on the learning of mathematics. This is because motivation is context-dependent. This means that individuals can have different types of mindsets and achievement goals depending on the contextual situation. For example, the same individual may have different mindsets and achievement goals in learning mathematics vs. participating in sports activities. Mathematics is chosen as the context in this study because there have been various reports on the motivational issues of lower-progress students in mathematics, a subject seen as cognitively demanding and anxiety-inducing for many students, in areas such as intrinsic motivation, mathematics value, mathematics enjoyment, and mathematics confidence (e.g., Herges et al., 2017; O’Shea et al., 2017). Research has highlighted that implicit theories of intelligence can have particular importance in challenging academic situations (Costa and Faria, 2018).

**Figure 1:**
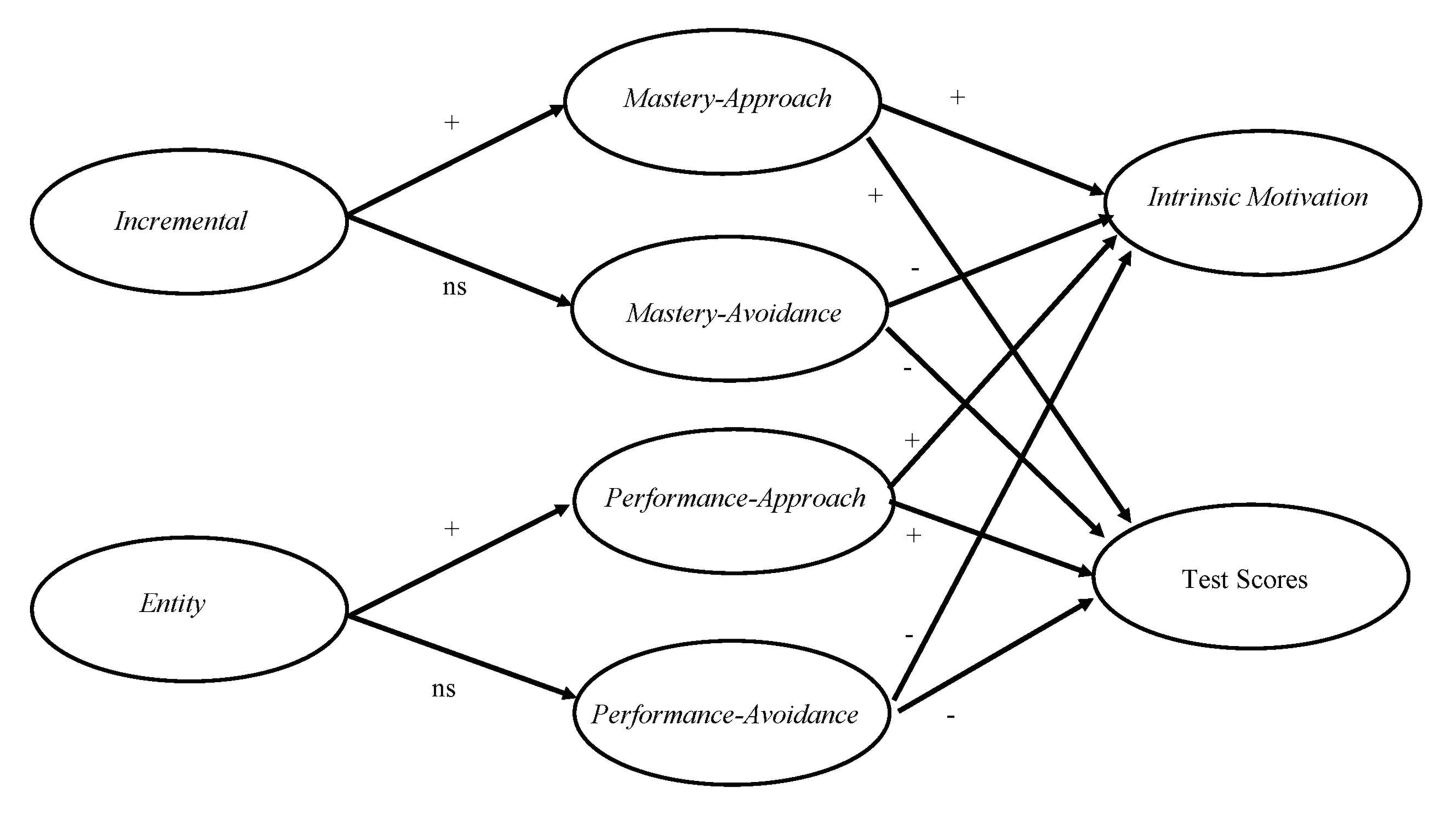
Hypothesized model of the relationships between mindsets, achievement goals, intrinsic motivation, and test scores.

In summary, Implicit Theories of Intelligence (Dweck, 2000) and 2 × 2 Achievement Goal Theory (Elliot and McGregor, 2001) have provided insights into the nature and antecedents of motivation and achievement. Very few studies have examined the underlying mechanisms between mindsets, achievement goals, and outcomes. In the domain of sports, some researchers (Wang and Biddle, 2001, 2007; Biddle et al., 2003) have shown that incremental beliefs and mastery goals are linked to intrinsic motivation and adaptive motivational patterns. In contrast, entity beliefs and performance goals are associated with low intrinsic motivation and maladaptive learning outcomes. Building on these findings, it was hypothesized that (H1) incremental beliefs would predict mastery-approach goals but not mastery-avoidance goals, (H2) entity beliefs would predict performance-approach goals but not performance-avoidance goals, (H3) mastery-approach and performance-approach goals would positively predict intrinsic interest and test scores, and (H4) mastery-avoidance and performance-avoidance goals would negatively predict intrinsic interest and test scores (see Figure 1).

Bearing in mind Van Yperen et al.’s (2014) finding on the moderation effect of nationality, we believe that in the current sample, mastery-approach goal would be a relatively strong positive predictor of performance (H3), while mastery-avoidance goal would be a relatively strong negative predictor of performance (H4). Considering that we are looking at lower-progress learners who may have low perceived competence, it is possible that incremental belief may predict mastery-avoidance goals (H1), while entity belief may predict performance-avoidance goals (H2).

## Materials and Methods

### Participants and Procedure

In this study, a sample of 1,201 lower-progress students from 17 Singapore secondary schools responded to the survey. There were 596 males and 580 females, and 25 of them did not state their gender. The students’ ages ranged from 13 to 17 years old (*M* = 14.68, *SD* = 0.57).

Prior to data collection, ethical approval was sought from the university’s Institutional Review Board and permission to collect data from schools was obtained from the Ministry of Education (Singapore) and the respective school principals. The heads of mathematics department were then contacted to arrange a time slot for the administration of the questionnaire. Before responding to the questionnaire, students provided consent after having been informed of the nature of the research project, that participation in the study was voluntary, that they could withdraw at any time, and that their confidentiality would be maintained. The students took less than 15 min to complete the survey under classroom conditions.

### Measures

#### Implicit Theories of Intelligence Scale

The six-item Implicit Theories of Intelligence Scale (Dweck, 2000) was used to assess students’ mindsets. Three items each were used to measure entity mindset (e.g., “I have a certain amount of intelligence, and I really cannot do much to change it”) and incremental mindset (e.g., “I can always greatly change how intelligent I am”). Responses were given on a 6-point Likert scale (1 = *Strongly agree*, 6 = *Strongly disagree*).

#### Achievement Goal Questionnaire

The Achievement Goal Questionnaire (Elliot and McGregor, 2001) was used to assess the four types of achievement goals with three items per subscale. In this study, the items were adapted to reflect the context of mathematics learning. The four goals were mastery-approach (e.g., “I want to learn as much as possible from my mathematics class”), mastery-avoidance (e.g., “Sometimes I’m afraid that I may not understand the content of my mathematics class as thoroughly as I’d like”), performance-approach (e.g., “My goal in my mathematics class is to get a better grade than most students”), and performance-avoidance (e.g., “My goal in my mathematics class is to avoid performing poorly”). The items focused on the standard of competence per se, that is, task‐ and self-competence/incompetence for mastery-based goals and normative competence/incompetence for performance-based goals. Students indicated the extent to which they agreed that the statements were true in describing them on a 7-point Likert scale (1 = *Not true at all*, 7 = *Very true*).

#### Intrinsic Motivation Inventory

The interest subscale of the Intrinsic Motivation Inventory (IMI; McAuley et al., 1989) was adapted to assess students’ interest in learning mathematics (three items; e.g., “I think mathematics is quite enjoyable”). It is considered the self-report measure of intrinsic motivation. The items were rated on a 7-point Likert scale (1 = *Not true at all*, 7 = *Very true*).

#### Mathematics Performance

The teachers provided the students’ mathematics test scores as an outcome measure. The school-based tests were based on the national curriculum and were taken one to two months after completion of the survey. The possible test scores range from 0 to 100.

## Results

### Confirmatory Factor Analysis (CFA)

CFA was conducted to examine the measurement model with all the constructs directly estimated based on their items. There were seven latent factors with its indicators, and the two beliefs, two mastery goals, and two performance goals were allowed to be correlated. EQS for Windows 6.3 (Bentler, 2006) was used as the analysis tool for CFA and SEM. Goodness-of-fits of the model were assessed with the robust *χ*^2^ test statistics, the Bentler-Bonett normed fit index (NFI), the Bentler-Bonett non-normed fit index (NNFI), the comparative fit index (CFI), the mean square error of approximation (RMSEA), and its 90% confidence intervals. Typical cutoff scores taken to, respectively, indicate adequate and excellent fit to the data were used: (a) values greater than 0.90 and 0.95 for the NFI, NNFI, and CFI and (b) values smaller than 0.08 and 0.06 for the RMSEA (Hu and Bentler, 1999; Marsh et al., 2005). Results of the CFA showed an adequate fit for the measurement model (scaled *χ*^2^ = 947.47, *df* = 178, NFI = 0.928, NNFI = 0.930, CFI = 0.941, RMSEA = 0.053, 90% CI of RMSEA = 0.049–0.056). This provided adequate factorial validity to the measurement model.

### Descriptive Statistics

The means, standard deviations, internal reliabilities (rho; Fornell and Larcker, 1981), and latent correlations of the variables are presented in Table 1. The rho coefficients ranged from 0.73 to 0.92, indicating satisfactory internal reliabilities for all the subscales.

**Table 1:** Means, standard deviations, and internal consistencies for all variables.

		Mean	SD	Range	Rho	1	2	3	4	5	6	7
1.	Incremental	3.67	1.14	1–6	0.84	1.00						
2.	Entity	3.61	1.13	1–6	0.84	−0.12^**^	1.00					
3.	Mastery-approach	5.31	1.25	1–7	0.84	0.18^**^	−0.01	1.00				
4.	Mastery-avoidance	5.08	1.22	1–7	0.75	0.06^*^	0.04	0.59^**^	1.00			
5.	Performance-approach	4.64	1.52	1–7	0.86	0.13^**^	0.09^**^	0.45^**^	0.40^**^	1.00		
6.	Performance-avoidance	5.33	1.33	1–7	0.73	0.04	0.08^**^	0.48^**^	0.59^**^	0.46^**^	1.00	
7.	Intrinsic motivation	4.34	1.57	1–7	0.92	0.19^**^	−0.04	0.55^**^	0.22^**^	0.31^**^	0.14^**^	1.00
8.	Test scores	52.21	17.49	3–97	---	0.11^**^	0.01	0.17^**^	−0.01	0.17^**^	−0.01	0.40^**^

* *p* < 0.05.

** *p* < 0.01.

Essentially, the students had moderate incremental and entity beliefs (means = 3.67 and 3.61 respectively) (using means ≥ 4.5 on the seven-point scale as high, as suggested by Liu et al., 2009) and relatively high achievement goals (4.64 ≤ mean ≤ 5.33). They also reported moderate intrinsic motivation (interest) in mathematics (mean = 4.34). The mathematics test scores ranged from 3 to 97 marks with a mean of 52.21 and *SD* = 17.49 and were largely normally distributed (skewness = −0.18, *SE* = 0.07; kurtosis = − 0.43, *SE* = 0.14). The correlations among the measures indicate that all four achievement goals were positively associated (0.40 ≤ *r* ≤ 0.59).

### Structural Equation Modeling

**Figure 2:**
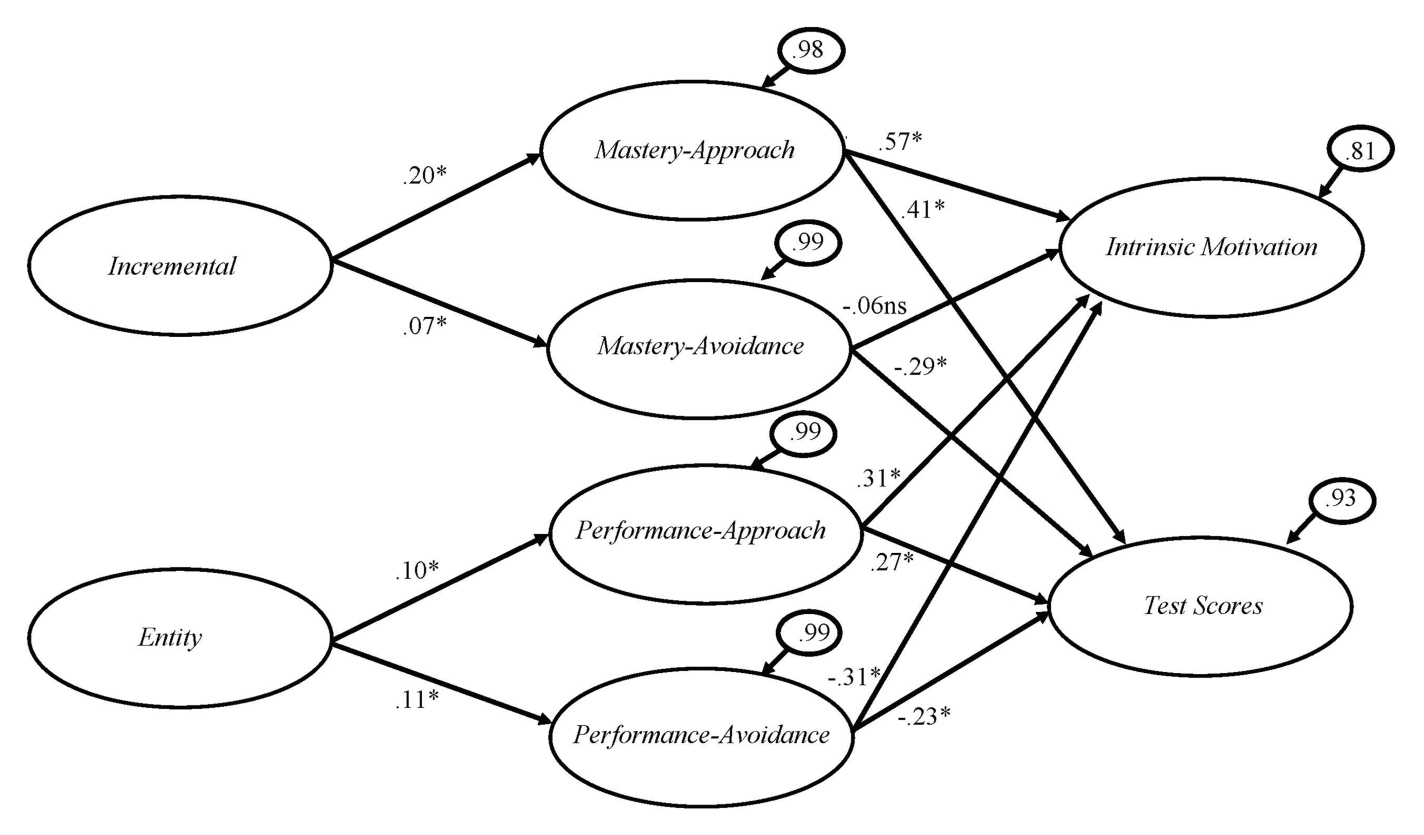
Standardized solution of the hypothesized model. ^*^p < 0.05.

Before conducting the SEM, the intraclass correlations (ICC) of the main variables with school as a grouping variable were computed. It was found that the mean ICC was 0.019, representing less than 2% of the variance which was attributed to the school membership; thus, multilevel analysis was not conducted. The results of the structural equation modeling with full latent model indicated a good fit of the model to the data (robust *χ*^2^ = 1154.08, *df* = 196, NFI = 0.995, NNFI = 0.995, CFI = 0.996, and RMSEA = 0.066, 90% CI of RMSEA = 0.062, 0.070). Figure 2 shows the standardized solution of the hypothesized model. It can be seen that incremental mindset predicted mastery-approach goal and, in turn, predicted intrinsic motivation and mathematics test scores. Mastery-avoidance goal did not predict intrinsic motivation but was negatively associated with mathematics test scores. Entity mindset predicted performance-approach and performance-avoidance goals. Performance-approach goal was positively linked to intrinsic motivation and mathematics test scores; performance-avoidance goal, however, negatively predicted intrinsic motivation and mathematics test scores. The model accounted for 35.9% of variance in intrinsic motivation and 13.8% in mathematics test scores.

## Discussion

Motivation is every educator’s business. It is of particular concern to educators of lower-progress students who often have to innovate on pedagogical practices and expend extra effort in engaging their students to learn. This study sought to identify the predictors of lower-progress students’ intrinsic motivation (interest) and achievement in mathematics in Singapore. In doing so, this study hopes to offer suggestions for intervention that can promote learning engagement and academic performance in the classrooms of lower-progress students. More specifically, this study sought to examine the influence of mindsets (incremental and entity) and achievement goals (mastery-approach, mastery-avoidance, performance-approach, performance-avoidance) on lower-progress students’ intrinsic motivation (interest) and academic performance (score).

The descriptive statistics showed that the scores for entity and incremental mindsets were moderate, that is, the students did not endorse any particular mindsets strongly, which is consistent with Burnette et al.’s (2013) observation. However, the two mindsets were barely correlated. This means that the two-belief system may lead to different processes.

Dweck and her colleagues (Dweck et al., 1995; Dweck, 2000) had postulated that mindsets play an important role in academic learning. Between the two mindsets—incremental vs. entity that individuals adopted—incremental mindset has been observed to be more adaptive. Relative to the entity mindset, the incremental mindset had consistently predicted higher interest (e.g., Dweck, 1986; Ng, 2018) and better academic achievement (e.g., Blackwell et al., 2007; Bostwick et al., 2017). Dweck and her colleagues (Dweck and Leggett, 1988; Dweck, 2000) also expounded that mindsets set up both a motivational and cognitive framework that affects individuals’ beliefs in and responses to achievement situations. To this end, individuals with incremental mindset with its belief in the malleability of intelligence and the importance of effort and growth should facilitate the adoption of mastery goals. In contrast, individuals with entity mindset with its belief in a fixed level of intelligence and that it is innate talent and not effort that defines success should facilitate the adoption of performance goals. Insofar that mindsets (incremental and entity) could trigger the adoption of different achievement goals (mastery-approach, mastery-avoidance, performance-approach, performance-avoidance), it would be reasonable to expect that the mindsets and achievement goals predict intrinsic motivation (interest) and academic performance (score) differently.

SEM was used to examine the relationships between mindsets, achievement goals, and outcomes in the current study. The results partially supported the first two hypotheses in that incremental mindset predicted mastery-approach goal and entity mindset predicted performance-approach goal. However, there were also significant positive relationships between incremental mindset and mastery-avoidance goal and between entity mindset and performance-avoidance goal. The findings could be due to the moderating effect of perceived competence suggested by Dweck (2000) and Wang et al. (2009). In essence, Wang et al. (2009) found that in the domain of sports, among students with high perceived competence, entity mindset did not lead to performance-avoidance goal, but when perceived competence was low, entity mindset positively predicted performance-avoidance goal. Similarly, when perceived competence was low, incremental mindset also predicted mastery-avoidance goal. Considering that the participants of the current study were lower-progress students who were likely to have low perceived competence, the rationalization seems logical. Nonetheless, there is a need for more empirical work with high-progress students as a comparison group to have a better understanding of the relationships between mindsets and achievement goals.

The results of the current study supported the third and fourth hypotheses in that mastery-approach and performance-approach goals positively predicted intrinsic motivation and test scores, while mastery-avoidance and performance-avoidance goals negatively predicted intrinsic motivation and test scores, albeit the path between mastery-avoidance and intrinsic motivation was not statistically significant. The findings are consistent with that of previous studies. For example, Biddle et al. (2003) reported that mastery-approach goals were positively related to enjoyment and intrinsic motivation, and Van Yperen et al.’s (2014) and Burnette et al.’s (2013) meta-analyses established that mastery-approach and performance-approach goals were related to positive performance attainment. Earlier research in the Singapore context found that students who were high in all four goals and those who were high only in mastery-approach goals tended to be associated with positive psychological characteristics and outcomes (e.g., Liu and Wang, 2005; Wang et al., 2007; Liu et al., 2009; Jang and Liu, 2012). In view of the current findings, it is tenable that although the students who were high in all four goals were already among the “top performers” in the earlier studies, their high avoidance goals could have held them back from achieving their potential. Researchers and educators in Singapore may want to work with their students who are high in all four goals and examine whether there is any merit in intervention to lower their avoidance goals.

Our results seem to be in line with Van Yperen et al.’s (2014) finding regarding the moderation effect of nationality. In essence, in the current sample, mastery-approach goal was indeed a relatively strong positive predictor of performance, while mastery-avoidance goal was a relatively strong negative predictor of performance.

The current study did not examine direct relationships between mindsets and learning outcomes. Nonetheless, the indirect relationships are consistent with studies that had reported that incremental mindset relative to entity mindset predicted higher interest (e.g., Dweck, 1986; Ng, 2018) and better academic achievement (e.g., Blackwell et al., 2007; Romero et al., 2014; Bostwick et al., 2017), and lend further support to Costa and Faria’s (2018) meta-analysis finding that incremental mindsets are positively associated with students’ achievement in Asia. Regarding entity mindsets, Costa and Faria’s found that entity mindsets are not significantly associated with achievement in Asia, but we documented a small significant indirect relationship between entity mindset and achievement. Costa and Faria rationalized that the results obtained in Eastern continents might reflect cultural differences. They suggested that more collectivist societies might encourage students to value the learning process over academic achievement and focus less on individual results. In contrast, a “more academically and professionally competitive society in Europe” could influence the students’ perspectives of intelligence and lead them to prioritize individual outcomes and to value positive assessment over knowledge (Elliott and Dweck, 1988; Robins and Pals, 2002). In the same vein, Kim and her colleagues noted that belief in incremental mindset is largely seen as a valued goal of child socialization in East Asian cultures (Kim et al., 2017) and is consistent with the teachings of many Asian cultures, e.g., Confucianism, that emphasize the responsibility of children to persevere and underscore the duty of parents to teach children the value of hard work (Kim and Wong, 2002; Park et al., 2014). There is also evidence to suggest that Asian American students more often attribute success and failure to effort compared to their European American counterparts (Mizokawa and Ryckman, 1990). Individuals of Asian descent are also more likely to have a self-improving orientation (i.e., focus on weaknesses to improve the self) rather than a self-enhancing orientation (i.e., focus on talents and successes) as compared to their European-American counterparts (Heine et al., 2001).

In the current study, the correlations among the four achievement goals indicate that they were moderately associated (0.40 ≤ *r* ≤ 0.59). Despite the inter-factor correlations, each goal had different associations with interest and mathematics scores, suggesting that all four goals were operative in the Singapore mathematics setting and were distinct. Our inter-factor correlations are comparable to those reported by Liu et al. (2009) with Singaporean youths in the academic setting. The finding is similar to Lau and Lee’s (2008) observation that performance-approach and performance-avoidance goals are differentiable for their students in Hong Kong. However, Bong and colleagues (Bong, 2005; Bong et al., 2013) found that South Korean students were unable to reliably separate performance-approach and performance-avoidance goals. They rationalized that this might be due, in part, to the nature of South Korean schools, which strongly emphasize normative achievement and social comparison among students. They posited that in such a learning context, the desire to do better than others might be indistinguishable from the desire not to perform worse than others (Bong et al., 2013). More extensive studies are needed to have a clearer understanding of students’ ability to differentiate between different kinds of achievement goals in different cultural contexts and for lower‐ and higher-progress students.

The findings of this study have several implications and practical applications. Given that incremental mindset was a much stronger predictor of learning outcomes compared to entity mindset, pedagogical effort should focus on inculcating incremental mindset in our students. This means that educators should imbue in students the value that intelligence and abilities are malleable and can be developed through effort and hard work. By attributing intelligence and abilities to effort and hard work, educators are empowering the students and conveying a message of hope and potential for the low-progress students to succeed in tasks. This value should be instilled in the students at a tender age so that it can be integrated and internalized into the students’ belief systems (Dweck, 2000).

For the older students, Dweck (2000) proposed the use of intervention to change mindset. In intervention, educators can explicitly teach students about incremental mindset, to attribute failure to a lack of effort rather than a lack of ability, to see failures as opportunities for self-reflection, self-improvement, and growth, and to embrace challenges. Educators can also provide more process praises (such as praises for effort or strategy) instead of praise for intelligence, give more encouragement and support (such as telling a student that he/she could improve with hard work), and suggest concrete strategies for improvement (such as telling a student that he/she needs to change his/her study strategies; Dweck, 2008). In addition, educators can share stories of mathematics greats as people who loved and devoted themselves to mathematics instead of being born geniuses (Good et al., 2007). In the same vein, educators can refrain from conveying the message of an entity mindset. This means to avoid telling students that talent alone leads to success, as doing so may discourage students from trying and may lead to learned helplessness and avoidance of challenges (Dweck, 2000). Empirically, studies have shown that such intervention studies are efficacious and that mindsets can be successfully primed to result in changes in the belief systems (e.g., Spray et al., 2006; Blackwell et al., 2007; Burnette and Finkel, 2012). Nonetheless, more empirical work needs to be done to understand the efficacy of such interventions, perhaps particularly in the Asian context.

From another perspective, educators may want to strive to increase students’ perceived competence so that they are more likely to adopt approach goals, regardless of their mindsets and/or stream membership. Competence can be developed through the provision of support structure and success experience (Reeve, 2016). Educators can create opportunities for students to experience success through bite-size mathematics assessments which are manageable for the students.

Considering that mastery‐ and performance-approach goals significantly and positively predicted learning outcomes, pedagogical interventions can also target at developing approach goals. In nurturing mastery-approach goal, educators can encourage their students to work on mastering their knowledge and skills and to focus on learning and self-improvement (Elliot and McGregor, 2001). The TARGET framework originally proposed by Epstein (1988) and Ames (1992) is relevant in creating a mastery climate in the classroom. *TARGET* is the acronym for *T*ask, *A*uthority, *R*ecognition, *G*rouping, *E*valuation and *T*ime (see Deemer, 2004, for details). For example, to promote mastery-approach goals and develop competence, teachers should design mathematics tasks so that they are purposeful, challenging, and varied. They should respond to students’ struggles with appropriate scaffolding and convey to them that learning requires effort and that mistakes are part of the experience. In addition, teachers can help students develop a sense of personal control and independence by giving them choices and involving them in decision-making when possible. When assessing and evaluating students’ work, the emphasis should be self-referenced, rather than norm-referenced. It should focus on individual progress and improvement.

Interestingly, the sense of competition and a desire to do better than others, which is summarized as performance-approach goal (Elliot and McGregor, 2001), can also be a driving force to better learning outcomes. While the findings from this study suggest that performance-approach goal could be adaptive, it has to be noted that mastery-approach goal has stronger effects on learning outcomes in comparison with performance-approach goal. It is also important to be aware that performance-approach goal may trigger negative emotions such as anxiety, worry, and negative affect (e.g., Elliot and McGregor, 2001; Jang and Liu, 2012). Thus, while educators can consider instilling a sense of healthy competition among the students, they should also advise the students that outperforming others should not be the only emphasis when learning.

Lastly, in line with previous studies (e.g., Burnette et al., 2013; Van Yperen et al., 2014), mastery‐ and performance-avoidance goals significantly and negatively predicted learning outcomes. This means that the adoption of mastery-avoidance goal (which involves avoiding challenging tasks) and performance-avoidance goal (which involves avoiding failure in front of others) can be detrimental to intrinsic motivation and mathematics performance. Fortunately, avoidance goals can be changed or lessened. Research studies have suggested that avoidance goals can be significantly reduced *via* purposefully designed interventions (e.g., Schnelle et al., 2010; Wang et al., 2018). For example, Wang et al. (2018) reported that individuals’ avoidance goals can be changed by directly targeting at participants’ understanding of avoidance goals and their detrimental effects on learning outcomes, and the deliberate adoption of more adaptive goals and behaviors until they become second-nature. As another example, through experimental manipulations, Schnelle et al. (2010) showed that the availability of goal-relevant resources such as time for learning, family support, close friends, and self-confidence could lessen the adoption of avoidance goals and to promote the adoption of more approach goals. Of note, even the perception on the availability of resources could influence the students’ goal adoption. Considering that some of the “top performers” in Singapore may be high in all four goals (e.g., Liu and Wang, 2005; Wang et al., 2007; Liu et al., 2009; Jang and Liu, 2012), which could be holding them back, more need to be done to lower students’ avoidance goals. Considering that students with low perceived competence have a higher tendency to adopt avoidance goals, such interventions may be more crucial for lower-progress learners as compared to their higher-progress counterparts.

In conclusion, using Implicit Theories of Intelligence (Dweck, 2000) and 2 × 2 Achievement Goal Theory (Elliot and McGregor, 2001), this study attempted to identity the predictors of intrinsic motivation (interest) and mathematics performance among a group of lower-progress students in Singapore. Findings from the present study suggest that the adoption of an incremental mindset and approach goals—mastery and performance—are beneficial for learning outcomes. For educators, a two-pronged approach—the nurturance of an incremental mindset and mastery- and performance-approach goals—would be useful for the promotion of intrinsic motivation and academic performance. Finally, the study adds to the literature done in the Asian context and lends support to the contention that culture may affect students’ mindsets and adoption of achievement goals, and their associated impact on achievement outcomes.

## Limitations of Study

Despite the interesting findings, the present study has its limitations. First, the study is cross-sectional in design and thus causality cannot be inferred, unless a number of conditions are fulfilled (e.g., Pearl, 2009; Grosz et al., 2020, March 18). For instance, Grosz et al. (2020, March 18) mentioned the need to (i) articulate a clear causal question and state the precise definition of the causal effect of interest; (ii) think carefully about how other variables relate to the treatment variable and outcome variable to identify potential confounders, colliders, mediators, and instrumental variables; (iii) establish an identification strategy and estimate the causal effect; and (iv) test the identification strategy against violations of assumptions to see how much the effect estimate would change if certain assumptions were violated. Alternatively, an experimental study can be conducted to test the causal relationships. Taking inspiration from an experimental study conducted in the domain of sports, an experiment can be set up where students are randomly assigned to one of three groups: entity mindset manipulation, incremental mindset manipulation, or a control group with no mindset manipulation (Spray et al., 2006). By examining students’ mindsets and achievement goals before and after the mindset manipulations, and their performance in a mathematics task, e.g., solving mathematics puzzles with increasing levels of difficulties, it will be possible to determine the causal relationships between mindsets, achievement goals, and performance.

Second, as with all self-report studies, the findings from this study might not be an accurate representation of the actual situations. Further studies using other research methodologies such as behavioral observations can be conducted to triangulate the findings. Third, this study was conducted with lower-progress stream students. Hence, the findings may not be generalized to students in the general population. It would be beneficial to replicate the study with students from different ability streams in Singapore or do a comparison study between higher- and lower-progress students to better understand the relationships between mindsets, achievement goals, and students’ intrinsic motivation and achievement in mathematics.

## Data Availability Statement

The raw data supporting the conclusions of this article will be made available by the authors, without undue reservation.

## Ethics Statement

The studies involving human participants were reviewed and approved by Nanyang Technological University Institutional Review Board (NTU-IRB), Nanyang Technological University, Singapore (IRB-2013-01-009). Written informed consent to participate in this study was provided by the participants’ legal guardian/next of kin.

## Author Contributions

WL carried out the research with the research team. She conceptualized and wrote the manuscript.

### Conflict of Interest

The author declares that the research was conducted in the absence of any commercial or financial relationships that could be construed as a potential conflict of interest.
